# Childhood trauma and depressive symptoms among orphaned children in Rwanda: parallel mediation by perceived social support and loneliness

**DOI:** 10.1186/s13034-025-00977-3

**Published:** 2025-11-24

**Authors:** Justin Singirankabo, Japhet Niyonsenga, Aristide Rutayisire Kibaki, Jeanne Marie Ntete, Jean Mutabaruka

**Affiliations:** 1 Department of Psychiatry and Behavioral Science, College of Medicine and Health Sciences, University of Rwanda, Kigali, Rwanda; 2 Department of Humanities, Social Sciences, and Cultural Industries, University of Parma, Parma, Emilia-Romagna, Italy; 3 Université de Picardie Jules Verne (UPJV), Amiens, France

**Keywords:** Childhood trauma experiences, Depression, Orphaned children, Perceived social support, Loneliness, Rwanda

## Abstract

**Background:**

Childhood trauma experiences are widely linked with depressive symptoms, especially among orphaned children in low-resource settings like Rwanda. These children often face multiple adversities, increasing their vulnerability to mental health issues. However, the mechanism underlying this relationship remains unclear.

**Objectives:**

The purpose of this study was to identify the mediating role of perceived social support and loneliness in the relationship between childhood trauma and depressive symptoms.

**Method:**

A cross-sectional study was conducted on 417 orphaned children aged between 10 and 18 years (M: 13.6, SD: 2.65), conveniently selected from the Gasabo district. Data were collected using standardized instruments to assess loneliness, perceived social support, depression symptoms, and childhood trauma. The data were analyzed using SPSS (Version 29.2); with mediation analyses performed using the PROCESS macro (Version 4.0).

**Results:**

Individual mediation analysis revealed that perceived social support and loneliness partially mediated the relationship between childhood trauma experiences and depressive symptoms. In the parallel mediation model, both perceived social support and loneliness fully mediated the relationship between childhood trauma experiences and depressive symptoms.

**Conclusion:**

The findings highlight the significant roles of perceived social support and loneliness as mediators in the relationship between childhood trauma experiences and depressive symptoms. The findings contribute to evidence-based interventions, informing mental health policies, therapeutic strategies, and social support programs tailored to orphaned children.

## Background

Childhood trauma experience refers to stressful and potentially traumatic events occurring from 0 to 17 years, these events may include emotional, physical, or sexual abuse; neglect; parental death or separation; exposure to domestic violence; and household instability [6, 22]. Globally, over a billion children are affected by violence each year, with many enduring multiple forms of trauma [13, 31]. Research by Merrick et al. [22] highlights the widespread prevalence of such adverse childhood experiences and their strong association with negative mental health outcomes, including depression and anxiety. Similarly, Chigiji et al. [6] emphasize the profound impact of these experiences on psychosocial development, particularly among children in vulnerable contexts.

Orphaned children, in particular, are highly susceptible to a range of adversities, including physical, emotional, and sexual maltreatment, parental loss, institutionalization, economic hardships, and neglect, which are associated with increased risk of depression, anxiety, post-traumatic stress disorder (PTSD), and other mental health issues [14, 23, 34].

Additionally, childhood trauma experiences are associated with lower levels of perceived social support, which can impair the ability to form and maintain healthy interpersonal and social relationships [7, 28]. Individuals with a history of childhood trauma are more likely to report lower perceived social support and higher reliance on negative coping strategies, both of which are linked to the development of major depressive disorder (MDD) [11, 17]. Childhood trauma can disrupt socio-emotional development, impair attachment and trust in caregivers, and reduce opportunities for forming supportive relationships [20]. These disruptions can diminish social competence and perceived social support, which in turn increase vulnerability to depressive symptoms [20].

In addition, loneliness plays a significant role in the development of depression among trauma-exposed children. Children who experience abuse, neglect, or parental loss are more likely to feel socially disconnected and lonely, which is a key predictor of depression [3, 4] and report higher levels of loneliness [11, 17]. Early adversity can impair a child’s ability to trust others and engage in social interactions, leading to prolonged social isolation and exacerbation of depressive symptoms [29, 32].

The intergenerational impact of the 1994 Rwandan genocide resulted in widespread trauma, loss of caregivers, and disrupted social networks, placing orphaned children at heightened risk of mental health problems [2, 26]. Understanding mechanisms such as perceived social support and loneliness in this context is therefore critical for informing interventions.

While several studies have examined the effects of childhood trauma on mental health [14, 23, 34], research associating childhood trauma and depressive symptoms among orphaned children is scarce. Furthermore, no studies have explored the mediating role of perceived social support and loneliness in the relationship between childhood trauma and depressive symptoms among orphaned children. Theoretically, the stress process model [25] suggests that stressors like trauma influence mental health outcomes through availability of coping resources such as social support. Likewise, attachment theory posits that early traumatic disruptions impair the ability to form healthy relationships; fostering loneliness and depression [10, 24]. And trauma theory [12, 15] explains how early life traumatic experiences disrupt psychological, emotional, and physiological development, leading to issues like depression, anxiety, and PTSD. It emphasizes the lasting impact of trauma on identity, safety, coping, and overall functioning; especially in vulnerable populations.

This study addresses this gap by identifying the relationship between childhood trauma experiences and depressive symptoms, and the potential mediating factors in this relationship among Rwandan orphaned children. We hypothesize that perceived social support and loneliness mediates the relationship between childhood trauma experiences and depressive symptoms. Specifically, we propose that lower perceived social support and higher loneliness due to childhood trauma are associated with depressive symptoms, while higher perceived social support can mitigate the negative effect of childhood trauma on depressive symptoms. This research innovatively examines multiple mediating variables, including perceived social support and loneliness, which have not been collectively studied in this context before; the study contributes to developing targeted interventions to mitigate the impacts of childhood trauma, informing both clinical practice and policymakers to support orphan children.

## Methodology

### Study design

This quantitative cross-sectional study design aimed to examine the mediating variables in the relationship between childhood trauma experiences and depressive symptoms among orphan children.

### Participants, sample size, and sampling techniques

The participants of this study consisted of children with orphan status living in the community of the Gasabo district. A sample size of 417 children with orphan status was selected using a convenient sampling technique because the study targeted orphaned children living in specific area of Gasabo district, which limited access to a broader population. This approach allowed us to efficiently recruit participants who met the inclusion criteria within available time and resource constraints. With the inclusion criteria of being single or double orphans, aged between 10 and 18 years old, and the exclusion criteria of having severe mental and intellectual disabilities. The sample size was determined using Cochran’s formula for sample size estimation, which is appropriate for large populations when the proportion of an attribute is unknown [8].

$$n=\frac{Z^{2}*P(1-P)}{e^{2}}$$

Using a 95% confidence level corresponding to 1.96 Z-score, a 5% margin of error, and an estimated proportion of 0.5 (for maximum variability), the initial sample size was calculated as 384 participants, assuming no response rate 10%, the final sample size was adjusted upward to 422. The response rate was at 98.8%.

### Data collection tools

#### Socio-demographic questionnaire

Participants completed a self-constructed questionnaire covering information on various factors, including age, sex, orphan status, educational level and performance, place of living, living conditions, and siblings’ status.

#### Childhood trauma questionnaire (CTQ)

The Childhood Trauma Questionnaire (CTQ) is a 28-item scale designed to assess childhood maltreatment across five subscales: physical, emotional, and sexual abuse, and physical and emotional neglect [1]. The questionnaire includes 25 items that evaluate various aspects of abuse. Additionally, the CTQ features a three-item minimization/denial scale to detect potential underreporting of traumatic experiences. Participants respond using a five-point Likert scale: (1) “Never true,” (2) “Rarely true,” (3) “Sometimes true,” (4) “Often true,” and (5) “Very often true.” Subscale scores range from 5 to 25, with classification guidelines provided in the CTQ scoring manual to determine levels of abuse and neglect: None, Moderate, Severe, and Extreme. In this study, the Cronbach’s alpha was 0.82. And a sample item is my family said hurtful or insulting things to me.

#### Multidimensional scale of perceived social support (MSPSS)

The multidimensional scale of perceived social support (MSPSS) is a 12-item scale designed to assess perceived support from three key sources: family, friends, and a significant other. Participants respond using a seven-point Likert scale, ranging from 1 (“Strongly Disagree”) to 7 (“Strongly Agree”). The MSPSS has a total score range of 12 to 84. In addition to these subscales, overall social support is also measured, with higher scores indicating greater perceived support [35]. To calculate the total support score, all 12 item responses are summed and then divided by 12. A mean score between 1 and 2.9 indicates low support; 3 to 5 suggests moderate support, and 5.1 to 7 represents high support [35]. In this study, the Cronbach’s alpha was 0.94. And sample item is there is a special person who is around when I am in need.

#### University of California, Los Angeles loneliness scale (UCLA loneliness Scale)

The UCLA Loneliness Scale is a 20-item designed to measure one’s subjective feelings of loneliness as well as feelings of social isolation [27]. Participants rate each item on a four-point scale, ranging from 1 (“Never”) to 4 (“Often”). However Items 1, 5, 6, 9, 10, 15, 16, 19, 20 are all reverse scored from 4 (“Never”) to 1 (“Often”). The overall score runs from 20 to 80, with higher levels denoting more severe symptoms of loneliness. In this study, the Cronbach’s alpha was 0.81. And a sample item is how often do you feel alone?

#### Center for epidemiological studies depression scale for children (CES-DC)

The CES-DC is a 20-item scale designed to assess depressive symptoms in children, with scores ranging from 0 to 60 [33]. The developers established a cutoff score of 15, suggesting the presence of depressive symptoms in children and adolescents [33]. Scores above this threshold may indicate significant depressive symptoms. Responses are recorded on a four-point Likert scale, ranging from 0 (“Not at all”) to 3 (“A lot”). However, items 4, 8, 12, and 16 are positively worded and are scored in reverse 3 (“Not at all”), 2 (“A little”), 1 (“Some”), and 0 (“A lot”). In this study, the Cronbach’s alpha was 0.88. And a sample item is: I wasn’t able to feel happy, even when my family or friends tried to help me feel better.

### Data analysis

The data analysis for this study was conducted using SPSS (Version 29.2) and the PROCESS macro (Version 4.0) by Hayes. Descriptive statistics were conducted to provide an overview of the sample’s socio-demographic characteristics and to evaluate the distribution of key variables. Pearson correlation analysis was conducted to explore bivariate relationships among childhood trauma experiences, depression symptoms, loneliness, and perceived social support. To assess mediation, PROCESS Model 4 was used to test two separate simple mediation models, where perceived social support and loneliness were examined as independent mediators of the childhood trauma experiences–depression relationship. A parallel mediation model was then conducted to assess the simultaneous mediating effects of both variables, with bootstrapped confidence intervals applied to determine statistical significance. Assumptions of normality, linearity, and multicollinearity were tested, and performed analyses of sensitivity to ensure findings’ robustness.

### Procedures

The Institutional Review Board of the College of Medicine and Health Sciences, University of Rwanda (Ref: CMHS/IRB/722/2024) approved the study, which was conducted following the ethical principles outlined in the Declaration of Helsinki. Permission was also obtained from the Gasabo District Administration (Ref. No 2198/07.01.02/2024). Informed consent and assent forms were obtained after explaining the study’s purpose to participants. All questionnaires were culturally adapted through translation and back-translation by independent psychologists. Participants were assured of voluntary participation, confidentiality, privacy, and respect. A trained psychologist was ready to provide emotional support to the participants who were psychologically distressed by the questionnaires. Data were collected through face-to-face administration of structured questionnaires in the participants’ respective schools and participants ‘home, to ensure a controlled and comfortable environment.

The questionnaires were administered in Kinyarwanda, the participants’ native language, after a rigorous translation and back-translation process to ensure accuracy and cultural appropriateness. Trained research assistants facilitated the process by reading items aloud to participants where necessary and providing clarification to ensure comprehension, especially for younger children.

On average, it took 15–25 min for each participant to complete the entire questionnaire. Data collection occurred in designated private spaces to maintain confidentiality and minimize distractions.

## Results

### Socio-demographic characteristics

As shown in Table 1, the sample comprised of 417 participants, with a nearly balanced gender distribution (46.4% male and 53.6% female). The majority identified as Christian (85.1%), followed by Muslim (14.4%), with very few reporting no religion (0.2%). About orphan status, almost half of the participants (49.0%) had lost both parents, while 20.9% had lost only their mother, and 30.0% had lost only their father. In terms of care background, most participants resided with relatives (41.4%), followed by foster care (35.9%), with fewer living with only their father (7.5%), only their mother (12.3%), or independently (2.9% in their own household). The majority (68.8%) had been in their current living arrangement for more than three years. Family connections remained strong, as 86.1% reported having siblings. Most participants (90.3%) were currently enrolled in school, with the majority attending primary school (73.5%), followed by secondary school (22.5%), and a small percentage in vocational training (4.0%). Among those not in schools, 91.9% cited financial reasons, with a minority reporting lack of interest (5.4%) or other reasons (2.7%). Academic performance varied, with 13.4% rating themselves as excellent, 39.4% as good, 37.0% as fair, and only 10.2% falling into the poor or very poor categories.

**Table 1:** Socio-demographic characteristics

Socio-demographic data		*N*	%
Gender	Male	193	46.4%
	Female	223	53.6%
Religion	Christianity	354	85.1%
	Islam	60	14.4%
	None	1	0.2%
	Other	1	0.2%
Orphan status	Both parents deceased	204	49.0%
	Only mother deceased	87	20.9%
	Only father deceased	125	30.0%
Care Background	Foster care	149	35.9%
	With relatives	172	41.4%
	With my father	31	7.5%
	With my mother	51	12.3%
	Own household	12	2.9%
Living arrangement period	Less than 1 year	25	6.1%
	1–3 years	104	25.2%
	More than 3 years	284	68.8%
Do you have siblings?	Yes	358	86.1%
	No	58	13.9%
Are you currently enrolled in school?	Yes	371	90.3%
	No	40	9.7%
Education level	Primary school	278	73.5%
	Secondary school	85	22.5%
	Vocational training	15	4.0%
If no, why are you not attending school?	Financial reasons	34	91.9%
	Lack of interest	2	5.4%
	Other	1	2.7%
Overall, how could you rate your academic performance?	Excellent	51	13.4%
	Good	150	39.4%
	Fair	141	37.0%
	Poor	29	7.6%
	Very poor	10	2.6%

### Correlation between variables

Our findings, as shown in Table 2, revealed that childhood trauma experiences were positively correlated with depression (*r* =.499, *p* <.001). Loneliness was strongly positively correlated with depression (*r* =.59, *p* <.001). In contrast, perceived social support was negatively correlated with depression (*r* = −.50, *p* <.001). Additionally, childhood trauma was negatively correlated with perceived social support (*r* = −.63, *p* <.001), and positively correlated with loneliness (*r* =.581, *p* <.001).

**Table 2:** Correlation between variables

Variables	CES-DC	PSS	CTQ	LC	
Depression (CES-DC)	–				
Perceived Social Support (PSS)	− 0.498^**^	–			
Childhood Trauma Questionnaire (CTQ)	0.499^**^	− 0.634^**^	–		
Loneliness (LC)	0.594^**^	− 0.391^**^	0.581^**^	–	

*Correlation is significant at the 0.05 level (2-tailed); **Correlation is significant at the 0.01 level (2-tailed)

### Individual mediation analysis of perceived social support in the relationship between childhood trauma and depression symptoms

**Fig. 1:**
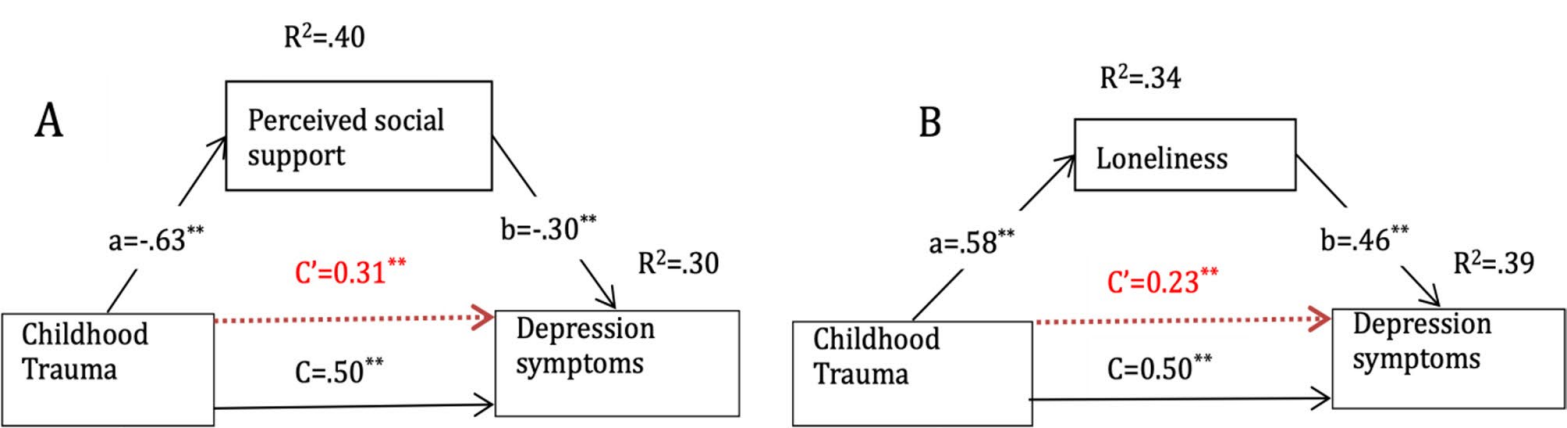
Panel (a) illustrates the mediation model ofperceived social support in the relationship between childhood trauma and depressive symptoms, withpath coefficients displayed for the direct effect (C′), indirect paths (a and b), and the explained variance(R^2^). Panel (b) illustrates the mediation model of loneliness in the same relationship, also presenting thepath coefficients for the direct effect (C′), indirect paths (a and b), and explained variance (R^2^)

Our findings in the mediation analysis revealed that perceived social support significantly mediates the relationship between childhood trauma and depression symptoms (Fig. 1A). Childhood trauma was a significant negative predictor of perceived social support (B = − 0.83, β=−0.63, SE = 0.049, *p* <.001, 95% CI [− 0.91, − 0.72]), explaining 40.26% of the variance. Furthermore, perceived social support negatively predicted depression symptoms (B = − 0.12, β=−0.30, SE = 0.022, *p* <.001, 95% CI [− 0.16, − 0.081]), explaining 30.4% of the variance after controlling for childhood trauma. The direct effect of childhood trauma on depression symptoms was significant (B = 0.16, β = 0.31, SE = 0.027, *p* <.001, 95% CI [0.11, 0.21]), but the total effect was stronger (B = 0.26, β = 0.50, SE = 0.0224, *p* <.001, 95% CI [0.22, 0.30]), indicating partial mediation. The indirect effect of childhood trauma on depression symptoms via perceived social support was also significant (B = 0.102, β = 0.193, SE = 0.0204, 95% CI [0.0625, 0.14]). These results suggest that perceived social support partially mediates the relationship between childhood trauma and depressive symptoms, accounting for 39.23% of the total effects.

### Individual mediation analysis of loneliness in the relationship between childhood trauma and depression symptoms

The mediation analysis also examined loneliness as a potential mediator in the relationship between childhood trauma and depression symptoms (Fig. 1B). Childhood trauma was found to be a significant positive predictor of loneliness (B = 0.40, β = 0.58, SE = 0.027, *p* <.001, 95% CI [0.34, 0.45]), explaining 33.74% of the variance. Additionally, loneliness was positively associated with depression symptoms (B = 0.347, β = 0.46, SE = 0.036, *p* <.001, 95% CI [0.27, 0.42]), explaining 38.87% of the variance after controlling for childhood trauma. The direct effect of childhood trauma on depression symptoms remained significant (B = 0.12, β = 0.23, SE = 0.024, *p* <.001, 95% CI [0.073, 0.17]), but the total effect was stronger (B = 0.26, β = 0.50, SE = 0.022, *p* <.001, 95% CI [0.22, 0.31]), demonstrating partial mediation. The indirect effect of childhood trauma on depression symptoms via loneliness was significant (B = 0.14, β = 0.27, SE = 0.020, 95% CI [0.102, 0.18]), with 53.57% of the total effect of childhood trauma on depression symptoms.

### Parallel mediation analysis of perceived social support and loneliness in the relationship between childhood trauma and depression symptoms

**Fig. 2:**
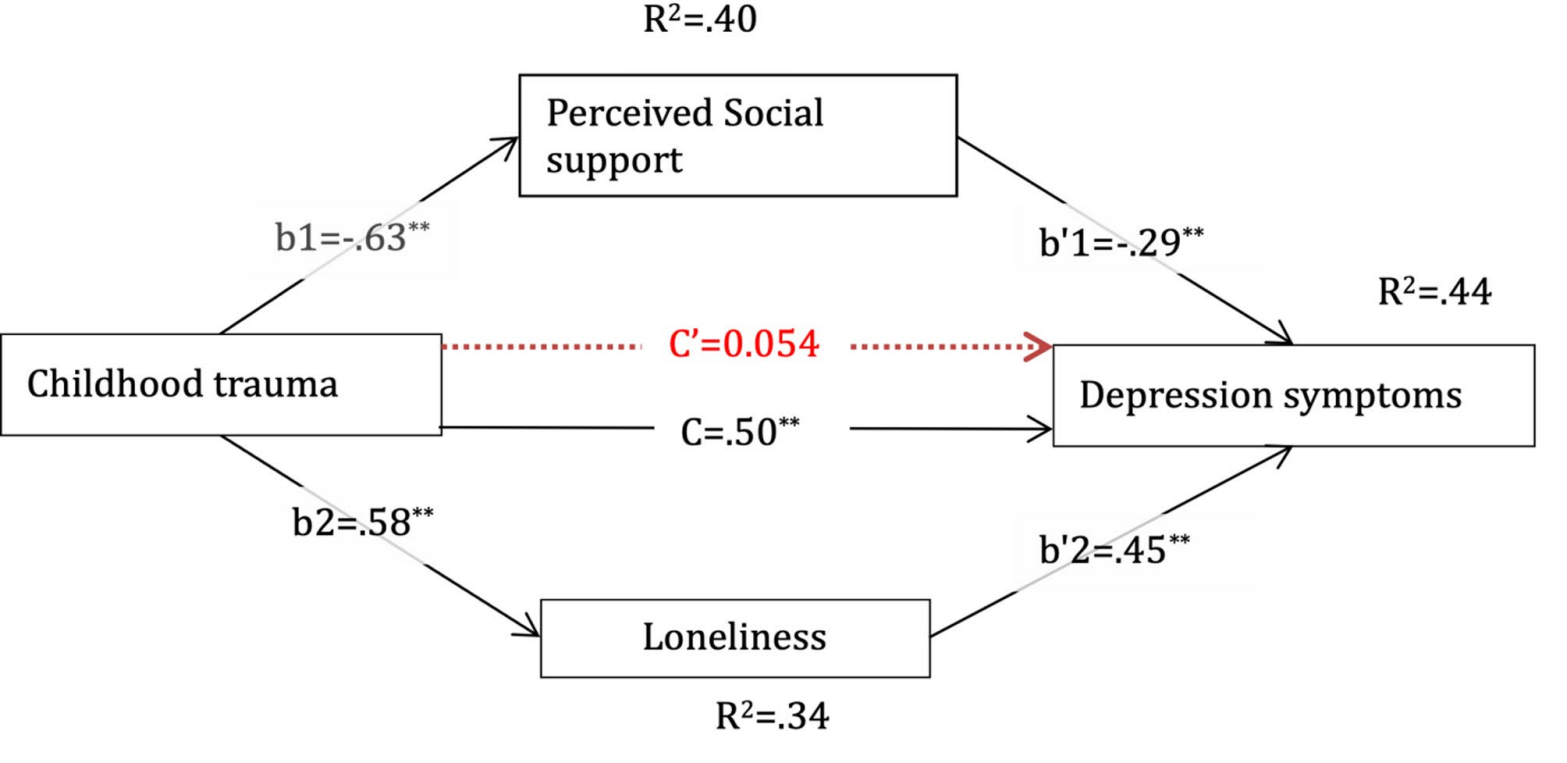
Parallel mediation analysis of perceived social support and loneliness in the relationship between childhood trauma and depression symptoms. This figure presents the standardized beta, and the broken red line shows the direct effect after accounting for mediators; **p-value ≤ 0.01

Inspired by individual mediation analysis, we explored a parallel mediation model with all mediators in the relationship between childhood trauma and depression symptoms (Fig. 2). The result of this study shows a correlation matrix displaying the relationships between key psychological variable-Depression (CESD), Perceived Social Support (PSS), Childhood Trauma (CTQ), and Loneliness (LC). The findings indicate that childhood trauma significantly predicted higher loneliness (B = 0.40, β = 0.58, SE = 0.027, *p* <.001, 95% CI [0.35, 0.45]) explaining 33.74% of the variance, and lower perceived social support (B = − 0.82, β=−0.63, SE = 0.05, *p* <.001, 95% CI [− 0.92, − 0.72]), explaining 40.26% of the variance. When predicting depression symptoms, loneliness was a significant positive predictor (B = 0.34, β = 0.45, SE = 0.034, *p* <.001, 95% CI [0.2730, 0.4078]). Similarly, perceived social support significantly reduced depression symptoms (B = − 0.12, β=−0.288, SE = 0.019, *p* <.001, 95% CI [− 0.155, − 0.078]). However, the direct effect of childhood trauma on depression symptoms became non-significant (B = 0.028, β = 0.054, SE = 0.028, *p* =.31, 95% CI [− 0.027, 0.084]), while the total effect remained significant (B = 0.26, β = 0.50, SE = 0.022, *p* <.001, 95% CI [0.22, 0.306]), indicating full mediation. The total indirect effect of childhood trauma on depression symptoms through both mediators was significant (B = 0.23, β = 0.44, SE = 0.026, 95% CI [0.18, 0.28]), accounting for 89.00% of the total effect. Specifically, loneliness accounted for a larger portion of the mediation effect (B = 0.14, β = 0.26, SE = 0.019, 95% CI [0.0997, 0.17]) than perceived social support (B = 0.096, β = 0.18, SE = 0.018, 95% CI [0.062, 0.13]). These findings suggest that the relationship between childhood trauma and depression symptoms is fully mediated by loneliness and perceived social support.

## Discussion

The primary objective of this research was to examine the mediating effects of perceived social support and loneliness in the relationship between childhood trauma experiences and depressive symptoms among orphaned children using both individual and parallel mediation analysis.

Findings from individual mediation analysis demonstrated that perceived social support partially mediated the relationship between childhood trauma experiences and depressive symptoms which aligns with existing literature suggesting that individuals with history of childhood trauma often struggle to develop and maintain supportive relationships, increasing their vulnerability to psychological distress [9, 16, 19]. This finding is consistent with the stress process model, which highlights how stressors like childhood trauma can lead to negative mental health outcomes, such as depressive symptoms, and how these outcomes are influenced by emotional and social resources, including social support [25, 30].

Similarly, loneliness was identified as a significant mediator between childhood trauma experiences and depressive symptoms. Aligned with previous literature suggesting that childhood adversity disrupts social development and increases the risk of social isolation, ultimately contributing to mental health struggles [29]. These findings are consistent with previous research indicating that loneliness significantly mediates the relationship between childhood abuse and various psychiatric outcomes [5, 11, 18, 21]. Findings from parallel mediation analysis revealed that loneliness and perceived social support acted as full mediators in the relationship between childhood trauma and depression. Aligned with the literature highlighted childhood trauma often disrupts early attachment bonds, leading to difficulties in forming and maintaining meaningful social relationships in adulthood; such disruptions contribute to persistent feelings of social disconnection and loneliness, which have been associated with an increased risk of developing depressive symptoms [3, 24]. Consistent with empirical evidence, individuals with a history of childhood trauma tend to report higher levels of loneliness and lower levels of perceived social support [11, 17].

### Findings implications and recommendations

The study found that childhood trauma significantly increases depressive symptoms among orphaned children, with social support and loneliness playing critical mediating roles. If these conditions persist, children face long-term challenges in emotional regulation, social interaction, and attachment formation, which heighten the risk of chronic mental health problems such as depression and anxiety. These difficulties can extend into adulthood, leading to poor academic achievement, limited employability, and socio-economic instability. However, early interventions that strengthen social support and reduce loneliness, such as peer support programs, community mentorship, and trauma-informed care can build resilience and promote positive development. Implementing such strategies is essential to mitigate the adverse effects of trauma and improve overall well-being.

The findings emphasize the need for policies that integrate mental health into child welfare systems, provide trauma-informed training for caregivers, and strengthen social support networks. Community-based interventions such as peer support and resilience building should be embedded within child protection programs. A multi-sectoral approach combining education, health, and community development is crucial to improve long-term well-being.

The findings of this study also have important implications for interventions and policies aimed at supporting orphaned children. First, programs that enhance perceived social support, such as peer support groups, mentorship programs, and caregiver training may help buffer the psychological impact of childhood trauma. Second, addressing loneliness through structured social activities, school-based counseling, and community engagement initiatives can reduce depressive symptoms and promote emotional well-being. Third, policymakers and NGOs should prioritize resources for mental health services within orphanages and community centers, ensuring that interventions are trauma informed and contextually relevant.

By addressing mediators like social support and loneliness, it is possible to break the link between early trauma and depression, fostering positive developmental outcomes for orphaned children.

### Strengths and limitations

Our study provides valuable insights into the individual and parallel mediation effect of perceived social support and loneliness in the relationship between childhood trauma experiences and depressive symptoms among orphaned children. The inclusion of multiple mediators provides a more comprehensive understanding of the mechanisms through which childhood trauma influences depressive symptoms. However, this study has some limitations.

First, the study employed a cross-sectional design, which restricts the ability to infer causality. Because all variables include childhood trauma, depressive symptoms, perceived social support, and loneliness were measured at the same point in time, the temporal order among them cannot be determined. This makes it unclear whether childhood trauma leads to depressive symptoms through perceived social support and loneliness, or whether depressive symptoms themselves influence perceptions of social support and feelings of loneliness, and also the reverse or bidirectional relationships cannot be ruled out. Future research should adopt longitudinal designs to establish temporal sequencing, strengthen causal interpretations, and to confirm the directionality of these associations. Second, the study did not examine other psychological mechanisms, such as coping strategies, emotion regulation, environmental stressors, and individual resilience factors. Including these aspects in future research would provide a more comprehensive understanding of the pathways linking childhood trauma to depression. Third, cultural and contextual factors, such as stigma, community support, and societal norms, were not considered in this study. These cultural and contextual factors may significantly influence how childhood trauma affects depressive symptoms and should be integrated into future research models. Fourth, this study used a convenience sampling technique, which may introduce selection bias and limit the generalizability of our findings. Future studies could employ probabilistic or stratified sampling methods to improve representativeness and minimize potential bias. Fifth, data in this study were collected using some self-report questionnaires such as socio-demographic questionnaire, which may be subject to possibility of reporting biases such as recall bias, social desirability bias, or misunderstanding of items, potentially affecting the accuracy of responses. Sixth, the study did not include clinical diagnostic interviews, which limits the ability to confirm depressive symptoms according to standardized psychiatric criteria.

## Conclusion

The findings from this study revealed significant relationship among key psychological variables: Childhood Trauma, Depression, Perceived Social Support, and Loneliness. Notably, a positive correlation exists between Depression and both Childhood Trauma and Loneliness. This implies that individuals experiencing higher levels of childhood trauma and loneliness are likely to report more severe depressive symptoms. Conversely, Perceived Social Support demonstrates a negative correlation with these factors, suggesting that stronger social support can mitigate the adverse effects of trauma and loneliness, acting as a protective buffer. Furthermore, the strong negative correlation between PSS and CTQ indicates that those with greater childhood trauma often perceive themselves as having less social support. Additionally, the link between CTQ and LC suggests that traumatic experiences can lead to increased feelings of loneliness later in life, highlighting the importance of fostering supportive relationships. Interestingly, the findings of this study provide strong evidence that loneliness and perceived social support mediate the relationship between childhood trauma experiences and depressive symptoms among orphaned children. The mediation model suggests that these factors partially explain how childhood trauma contributes to depression. These findings highlight the importance of addressing not only the traumatic experiences themselves but also the psychosocial factors that influence mental health outcomes. These findings underscore the need for multi-level interventions and the need to strengthen social support networks to mitigate the adverse mental health consequences of childhood trauma. For parents and caregivers, creating safe, supportive, and emotionally responsive environments is crucial to mitigating the effects of early trauma. To support the psychosocial well-being of orphaned children, schools and community centers should implement structured interventions that facilitate peer support, mitigate experiences of loneliness, and promote mentorship. Such programs are essential for fostering a sense of belonging and emotional security among vulnerable youth populations. NGOs and governments need to invest in trauma-informed policies and interventions, such as providing mental health services in child welfare systems, orphanages, training caregivers in emotional and social support strategies, and integrating psychosocial care into child welfare systems and implementing structured social support programs to help orphaned children cope with childhood trauma and reduce depressive symptoms. Overall, interventions that strengthen social connections, build resilience, and foster self-worth can substantially reduce depressive symptoms and promote long-term mental well-being in children exposed to early life adversities. Future policies and programs should prioritize these approaches to break the cycle of trauma and improve the quality of life for vulnerable children.

## Data Availability

No datasets were generated or analysed during the current study.
